# Artificial intelligence for medical imaging education: bibliometric and visual analysis

**DOI:** 10.3389/fmed.2026.1821176

**Published:** 2026-07-28

**Authors:** Yiman Wu, Wenqi Wu

**Affiliations:** 1 Medical Imaging Institute, Jiangsu Medical College, Yancheng, China; 2 School of Data Science and Artificial Intelligence, Wenzhou University of Technology, Wenzhou, China

**Keywords:** artificial intelligence, bibliometrics, cross-database validation, generative AI, medical imaging, radiology education

## Abstract

**Background:**

While bibliometric analyses have examined the adoption of Artificial Intelligence (AI) in general medical education contexts, the specific knowledge structures, collaborative networks, and evolutionary pathways of AI for medical imaging education remain poorly characterized quantitatively, particularly regarding the transition from technical feasibility to systematic pedagogical integration and cross-institutional partnerships.

**Methods:**

A structured literature search of English-language original articles and reviews published between 2006 and 2025 was performed in the Web of Science Core Collection (WoS) and Scopus, with the former serving as the primary database and the latter for external validation. Bibliometric analyses covering citation trends, collaboration networks, keyword bursts, and journal distributions were conducted using CiteSpace, VOSviewer, and Bibliometrix.

**Results:**

This search retrieved 577 articles from WoS, revealing exponential growth since 2019, with 226 articles published in 2025. These publications were contributed by 3,739 authors from 1,479 institutions across 80 countries/regions. Cross-database validation supported the consistency of these findings (Spearman r = 0.97, *p* < 0.001), with the United States (166 articles, 28.8%) and China (115 articles, 19.9%) as the primary contributors. BMC Medical Education, Insights into Imaging, and Academic Radiology were the most productive journals. Keyword analysis revealed diversification from foundational algorithms toward interactive, generative AI applications in medical imaging education, documenting an expanded scope integrating technical development with pedagogical innovation and ethical governance.

**Conclusion:**

This study characterizes a dual-polar production structure with the United States and China as dominant contributors, and a journal distribution spanning medical education and imaging technology venues. The post-2022 emergence of generative AI keywords documented a new thematic cluster alongside foundational machine learning research, indicating an evolving methodological repertoire. These findings provide a quantitative baseline for evidence synthesis in this interdisciplinary domain, with implications for curriculum development, algorithmic performance benchmarks, and ethical governance, subject to standard bibliometric data coverage and currency constraints.

## 1 Introduction

AI is increasingly reshaping medical imaging education, driving innovations ranging from automated case libraries to simulated diagnostic training (1, 2). While previous reviews (2019–2022) have predominantly examined AI adoption in general medical education contexts (3–5), recent scoping reviews guided by Best Evidence Medical Education (BEME) principles have systematically mapped the diverse applications of AI across medical education stages and specialties (6), and complementary systematic reviews have examined the specific impact of generative AI on health professional education (7). Technically, deep learning models have demonstrated potential to match experienced specialists’ diagnostic accuracy in skin cancer screening and oncologic imaging, providing foundations for standardized teaching case repositories (8, 9). Pedagogically, AI-assisted interpretation training, virtual patient simulation, and adaptive learning systems are reshaping residents’ differential diagnosis competencies (10, 11). Beyond imaging-specific applications, general-purpose large language models (LLMs) demonstrate clinical reasoning capabilities applicable to medical education broadly (12, 13). However, the specific knowledge structures, collaborative networks, and evolutionary pathways of AI for medical imaging education remain poorly characterized quantitatively, particularly regarding the transition from technical feasibility to systematic pedagogical integration and cross-institutional partnerships. In this context, generative AI, large language models (LLMs), and specific implementations such as ChatGPT and GPT-4 denote a hierarchical relationship wherein LLMs constitute a technical subclass of generative AI, and ChatGPT/GPT-4 represent specific commercial applications (12–14). Systematic reviews have mapped ChatGPT’s radiology applications (15, 16) alongside its limitations, including accuracy variability, hallucination risks (10, 17), and adverse cognitive effects of student over-reliance (18). Existing literature on AI in medical imaging education predominantly focuses on isolated dimensions, namely technical performance validation (19), stakeholder attitudinal surveys (14), ethical risk assessments (20, 21), or curriculum integration reports (22–24), which are examined in relative isolation from one another. Recent scholarship has proposed principle-based approaches to AI ethics education, extending traditional medical ethics principles to incorporate public health ethics considerations to address the dynamic challenges of AI integration (23, 24). In response, expert-opinion-based guidelines have advocated AI integration into medical imaging curricula to alleviate career anxiety among trainees (20, 21). Scoping reviews have cataloged diverse applications spanning personalized curriculum generation, diagnostic support tools, and automated evaluation systems (25). However, integrated quantitative analyses that simultaneously map knowledge structures, collaboration networks, and thematic evolution across the full temporal span of the field are notably absent. This lack of systemic integration risks producing siloed interventions that address single dimensions without accounting for their reciprocal influences. More critically, research on standardized assessment and technological boundaries specific to medical imaging education requires further consolidation. Benchmark evaluations such as MultiMedQA have highlighted the substantial discrepancy between breakthroughs in objective metrics and actual clinical reasoning capabilities (26). Recent longitudinal assessments of LLM performance in radiology have demonstrated substantial accuracy fluctuations over time, underscoring the need for continuous, radiology-specific standardized benchmarking metrics (27). Specifically, how deep learning diagnostic accuracy translates into standardized teaching case repositories, how reported trainee career anxiety informs curriculum design priorities, and how performance benchmarking tools mediate between algorithmic evaluation and educational assessment outcomes remain empirically unexamined.

Four specific gaps motivate this study, namely: (1) Publication dynamics: Bibliometric profiles of publication volume, citation trends, and growth trajectories in this field remain undocumented; (2) Collaboration patterns: Multi-level collaboration structures and their temporal evolution remain unmapped; (3) Thematic evolution: How research themes have evolved from traditional algorithmic approaches to emerging AI technologies remains unmapped through bibliometric methods; (4) Cross-database validation: The consistency of observed bibliometric patterns across independent indexing systems remains untested. Addressing these gaps is necessary to advance from fragmented studies toward integrated strategies for AI integration in medical imaging education.

To address these gaps, this study conducts a comprehensive bibliometric analysis (2006–2025) of English-language literature on AI in medical imaging education. Using WoS as the primary database and Scopus for external validation, the study examines publication trends and citation trajectories, country-level and institutional collaboration networks, journal distribution patterns and concentration metrics, keyword evolution and burst dynamics, and co-citation thematic structures. Cross-database validation assesses the consistency of observed patterns. Findings aim to provide an evidence-based baseline for strategic development in this interdisciplinary domain. This study builds upon prior fragmented analyses by revealing how publication dynamics, collaboration networks, and thematic shifts collectively characterize the field’s developmental trajectory, yielding integrative insights that single-dimension studies cannot capture. Cross-database validation further addresses whether these observed patterns are consistent with indexing system variations. By delineating the intellectual structure, global collaboration networks, and thematic evolution of this interdisciplinary domain, this study aims to inform curriculum developers, educational policymakers, and researchers seeking to integrate AI technologies responsibly into medical imaging training programs.

## 2 Materials and methods

### 2.1 Search strategy

This study employs bibliometric analysis rather than systematic review methodology. Bibliometric analysis examines publication patterns, citation networks, and thematic evolution through quantitative visualization, which differs from systematic review’s focus on evidence synthesis and quality assessment. Bibliometric data were retrieved from WoS and Scopus. Searches were conducted on February 15, 2026. In WoS, the search was conducted using the “Topic” (TS) field, which includes title, abstract, author keywords, and Keywords Plus. The query was: TS = (“artificial intelligence” OR “AI”) AND TS = (“medical imaging” OR “medical image*”) AND TS = (“educat*” OR “teach*”). In Scopus, the equivalent search was performed using TITLE-ABS-KEY, covering title, abstract, and author keywords: TITLE-ABS-KEY((“artificial intelligence” OR “AI”) AND (“medical imaging” OR “medical image*”) AND (“educat*” OR “teach*”)). Both searches were restricted to English-language articles published between January 1, 2006, and December 31, 2025. WoS served as the primary database for bibliometric analysis, while Scopus was used for external validation. Cross-database validation was performed to assess the consistency of findings across independent indexing systems. The “educat*” stem was selected to capture education-related terminology across title, abstract, and keyword fields, while avoiding the broad inclusion of terms such as “training” or “curriculum” without concurrent AI and medical imaging constraints, which risks capturing non-relevant literature. This precision-focused three-part search strategy prioritizes thematic coherence over recall sensitivity. This trade-off aligns with standard bibliometric practice, where meaningful network visualization requires a corpus with well-defined topical boundaries (28, 29).

A post-hoc sensitivity check was performed to assess the robustness of this retrieval strategy. An expanded Scopus search was conducted using alternative educational terminology (for example, “curriculum,” “training,” “radiology education”) within the same three-part framework: TITLE-ABS-KEY((“artificial intelligence” OR “AI”) AND (“medical imaging” OR “medical image*”) AND (“educat*” OR “teach*” OR “curriculum” OR “training” OR “radiology education”)). This expanded search retrieved a substantially larger corpus containing considerable noise, such as technical literature on machine learning model training, generic medical education content lacking AI or imaging specificity, and workforce development literature unrelated to the target domain. This substantially increased the burden of data cleaning and compromised the thematic coherence required for interpretable bibliometric analysis. Consequently, the precision-focused primary search strategy was retained as the analytical corpus, with these supplementary results corroborating its selection.

### 2.2 Eligibility criteria

This study applied explicit inclusion and exclusion criteria. Inclusion criteria were limited to English-language original articles and reviews focusing on AI applications in medical imaging education, published between January 1, 2006, and December 31, 2025. Exclusion criteria encompassed early access publications, meeting abstracts, editorials, conference proceedings, book chapters, letters, and retracted articles. Screening discrepancies were resolved through discussion; unresolved cases were referred to a third reviewer, with final arbitration by a senior radiologist-educator if consensus could not be reached.

### 2.3 Data extraction and analysis

Three complementary software platforms (CiteSpace v6.4. R1, VOSviewer v1.6.20, and R/Bibliometrix v4.4.2) were deployed in tandem to ensure methodological triangulation of the bibliometric analyses (30–32). This multi-tool strategy capitalizes on the complementary analytical strengths of each platform. Specifically, CiteSpace was utilized for temporal burst detection and structural variation analysis (30), VOSviewer for network topology visualization and co-occurrence mapping (31), and R/Bibliometrix for statistical validation and longitudinal thematic analysis (32).

The following standard bibliometric variables were extracted from each record: publication metadata, author and institutional information, geographic origin, keywords, and citation data. Analyses were structured around standard bibliometric dimensions. Network analyses were performed using VOSviewer (co-authorship, co-citation, and keyword co-occurrence mapping), CiteSpace (burst detection, reference co-citation clustering, and dual-map overlay), and R/Bibliometrix (descriptive statistics, thematic mapping, and longitudinal visualization) (32). These tools collectively enable triangulation of findings across network topology, temporal emergence, and statistical trends. All data were extracted from WoS and Scopus; therefore, ethical approval was not required.

## 3 Results

### 3.1 Global overview

**Figure 1:**
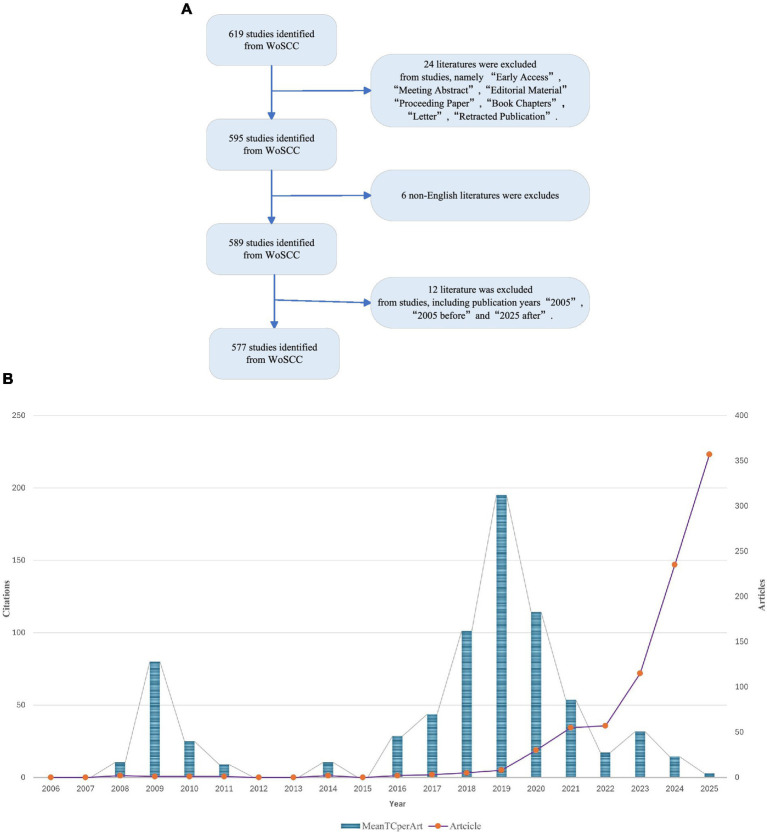
Bibliometric overview of AI for medical imaging education from WOS. **(A)** The flowchart of the search strategy of AI for medical imaging education from WoS. **(B)** Temporal trends in publications and citations (2006–2025). Mean citations per article were calculated as total citations divided by annual publication count.

Based on the flowchart of the search strategy (Figure 1A), a structured literature search of WoS (2006–2025) yielded 577 records for screening, comprising 432 original articles and 145 reviews. The average number of citations per article was 26.45. As a novel research field, 53 of these articles received over 50 citations. In total, 3,739 authors from 80 regions and countries, and 1,479 institutions, published related literature on AI for medical imaging education across 319 global journals. The reliability of these WoS-based metrics was supported through cross-database validation with Scopus.

### 3.2 Temporal trends in publications and citations

As illustrated in Figure 1B, the field exhibited distinct temporal trajectories for publication output (purple line) vs. citation impact (blue bars). Publication volume remained negligible (<5 articles annually) during 2006–2015, with sporadic single-digit outputs (e.g., 2 articles in 2008,1 in 2009–2011). Following minimal growth in 2016–2018 (2–4 articles annually), the field experienced a modest increase from 2019 (7 articles), accelerating to 23 articles in 2020 and surging to 78 articles in 2023, before reaching a historical maximum of 226 articles in 2025, with full-year indexing confirmed.

Conversely, mean citations per article displayed an inverse trend: an early peak in 2009 (80.00) driven by singular high-impact articles, and a historical maximum in 2019 (221.14) attributable to the high per-article citation impact during the low-output phase. Notably, as publication output expanded rapidly from 2020 onward, mean citations per article declined precipitously to 3.83 in 2025, a pattern consistent with emerging research domains where rapid publication expansion typically coincides with lower per-article citation rates, attributable to both volume distribution effects and the limited citation window for recent publications.

### 3.3 Distributions of countries/regions

Table 1 shows distinct productivity patterns. The USA leads with 166 articles (28.8%), followed by China (115, 19.9%). Together, they account for nearly half (48.7%) of total publications. Collaboration patterns reveal an inverse trend. Germany (48.1%) and the United Kingdom (45.2%) demonstrate the highest MCP percentages, significantly surpassing the USA (23.5%) and China (19.1%). The USA and China exhibit lower MCP percentages, while Germany and the UK demonstrate higher rates of international co-authorship. Notably, Canada (50.0%) exhibits the highest collaboration rate despite ranking sixth in total output. Research impact metrics show a different hierarchy. Germany (46.40) and Canada (35.00) achieve the highest average citations per article, contrasting sharply with lower-impact producers such as Saudi Arabia (5.70). Despite their substantial output volume, the USA (30.10) and China (21.20) exhibit relatively modest citation rates compared with higher-impact countries.

**Table 1:** Top 10 productive countries regarding study on AI for medical imaging education.

Rank	Country	Articles	Articles %	SCP	MCP	MCP %	Average article citations
1	USA	166	28.8	127	39	23.5	30.10
2	CHINA	115	19.9	93	22	19.1	21.20
3	UNITED KINGDOM	31	5.4	17	14	45.2	22.50
4	GERMANY	27	4.7	14	13	48.1	46.40
5	JAPAN	25	4.3	23	2	8.0	19.00
6	CANADA	20	3.5	10	10	50.0	35.00
7	ITALY	17	2.9	11	6	35.3	32.70
8	AUSTRALIA	16	2.8	9	7	43.8	18.90
9	SAUDI ARABIA	15	2.6	10	5	33.3	5.70
10	INDIA	13	2.3	9	4	30.8	22.50

SCP(single country publications); MCP(multiple country publications).

**Figure 2:**
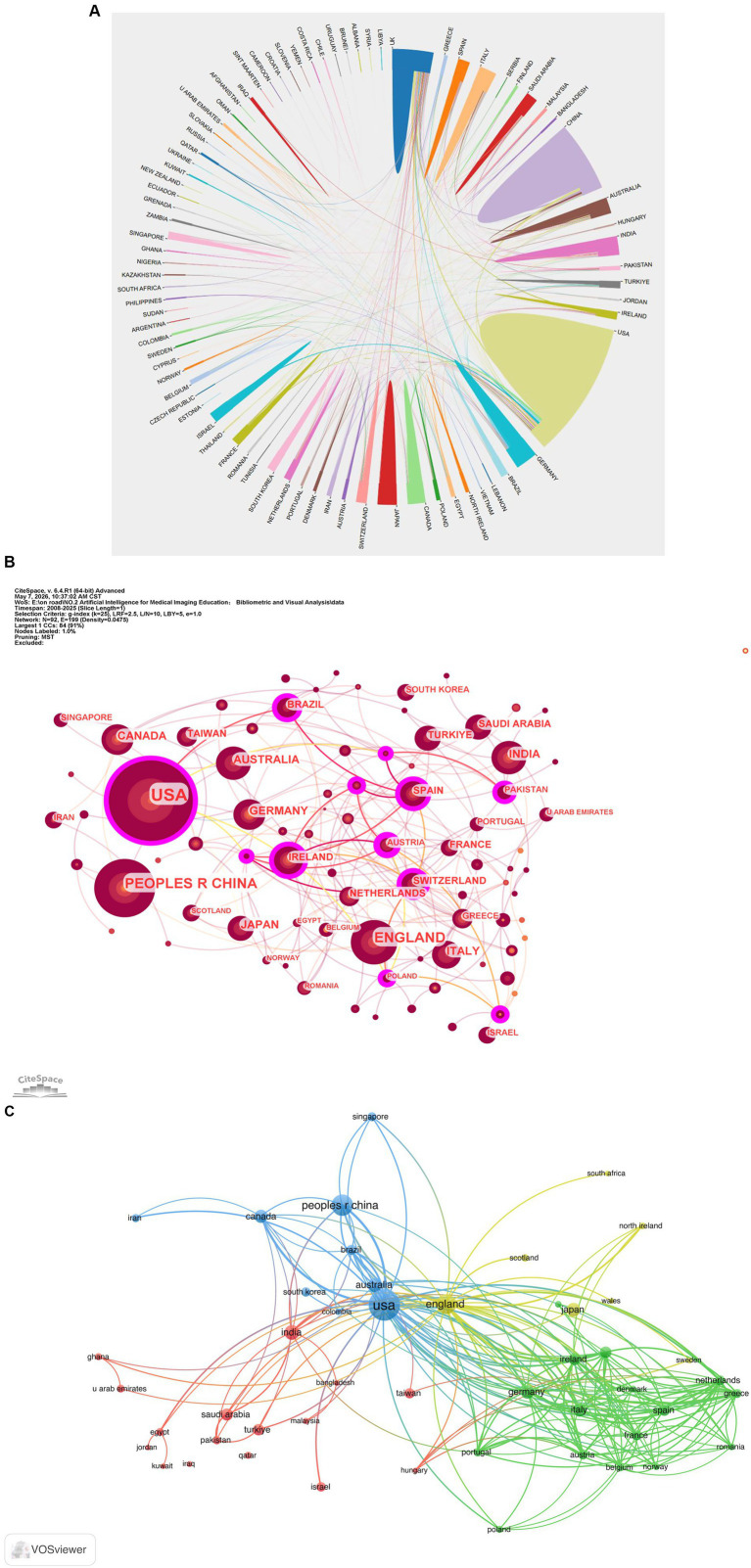
Geographic distribution and collaboration analysis. **(A)** Chord plot of country/region cooperation. **(B)** Global collaboration network on AI for medical imaging education. Node size represents publication output; purple rings indicate high betweenness centrality. **(C)** Global collaboration map of countries/regions.

Figure 2A presents the chord diagram of international collaboration networks in AI medical imaging education. Visually, the network exhibits a dual-polar structure with the USA and China occupying the two largest arc segments, with the former noticeably larger than the latter. Together, these two nodes account for most of the circle’s area. The UK and Germany exhibit dense chords connecting to multiple nations. Australia maintains collaborative ties with the USA and European partners. In contrast, China and Japan display thinner chords with fewer outgoing connections. These patterns correspond to the MCP data in Table 1: Germany (48.1%) and the UK (45.2%) demonstrate the highest international co-authorship rates, while China (19.1%) and Japan (8.0%) show lower rates.

Figure 2B highlights countries with high betweenness centrality (33), indicated by pink outer rings. These nodes occupy structural bridge positions (34). The USA connects the European cluster (England, Germany, Italy, and Switzerland) with the Asia-Pacific cluster (China, Australia, and Japan). England and India exhibit significant centrality relative to their publication volumes. Saudi Arabia, Austria, and Portugal also display distinctive centrality rings.

Figure 2C presents a global collaboration network algorithmically divided into seven clusters, with four dominant groups warranting particular attention. The green cluster centers on the USA as the largest node, encompassing Middle Eastern partners including Saudi Arabia, Egypt, and Qatar, alongside South Asian (Pakistan) and Scandinavian nations (Norway, Sweden). The yellow cluster is led by China, covering Asia-Pacific nodes (Singapore, Taiwan), Middle Eastern (Israel, Iran), and European (Scotland) partners. The blue cluster features England at its core, representing a densely interconnected Western European network spanning Switzerland, Austria, Belgium, Ireland, and Greece. The red cluster comprises Germany, France, Japan, South Korea, Spain, the Netherlands, and Brazil, occupying boundary positions between the American, European, and Asia-Pacific clusters. Dense inter-cluster connections link the USA (green) and England (blue). Significant linkages also connect the USA with China (yellow), while England maintains strong ties with Continental European nations in the red cluster. Red cluster nodes occupy boundary positions between the English-speaking and Asian networks, with Germany and France displaying connections to both clusters.

### 3.4 Distribution by institutions

Table 2 presents the leading institutions by article output. Harvard University (34 articles) and Harvard University Medical Affiliates (29 articles) occupy the top two positions, followed by the University of London (26 articles) and Harvard Medical School (24 articles). Centrality metrics reveal a contrasting pattern. Despite ranking second in output, Harvard University Medical Affiliates achieves the highest centrality (0.08), while Brigham & Women’s Hospital and Imperial College London, ranking ninth and tenth in output (14 and 13 articles, respectively), share the second-highest centrality (0.07). Temporally, six of the top 10 institutions first appeared in 2019 (Ranks 1–4, 6–7), with the remaining four distributed across 2020–2023 (Imperial College London in 2020; University College London and Brigham & Women’s Hospital in 2021; Mayo Clinic in 2023).

**Table 2:** Top 10 institutions by article output and corresponding centrality metrics.

Rank	Institutions	Count	Centrality	Year
1	Harvard University	34	0.06	2019
2	Harvard University Medical Affiliates	29	0.08	2019
3	University of London	26	0.04	2019
4	Harvard Medical School	24	0.03	2019
5	Mayo Clinic	21	0.03	2023
6	Stanford University	20	0.03	2019
7	University of California System	19	0.01	2019
8	University College London	14	0.03	2021
9	Brigham & Women’s Hospital	14	0.07	2021
10	Imperial College London	13	0.07	2020

i. “Harvard University” records listing “Harvard University” only, without any medical-school or hospital designation. ii. “Harvard University Medical Affiliates” records naming any Harvard-affiliated teaching hospital or clinical center. iii. “Harvard Medical School” records explicitly containing the phrase “Harvard Medical School.” iv. These categories represent overlapping organizational units within a single university ecosystem, rather than independent institutions.

**Figure 3:**
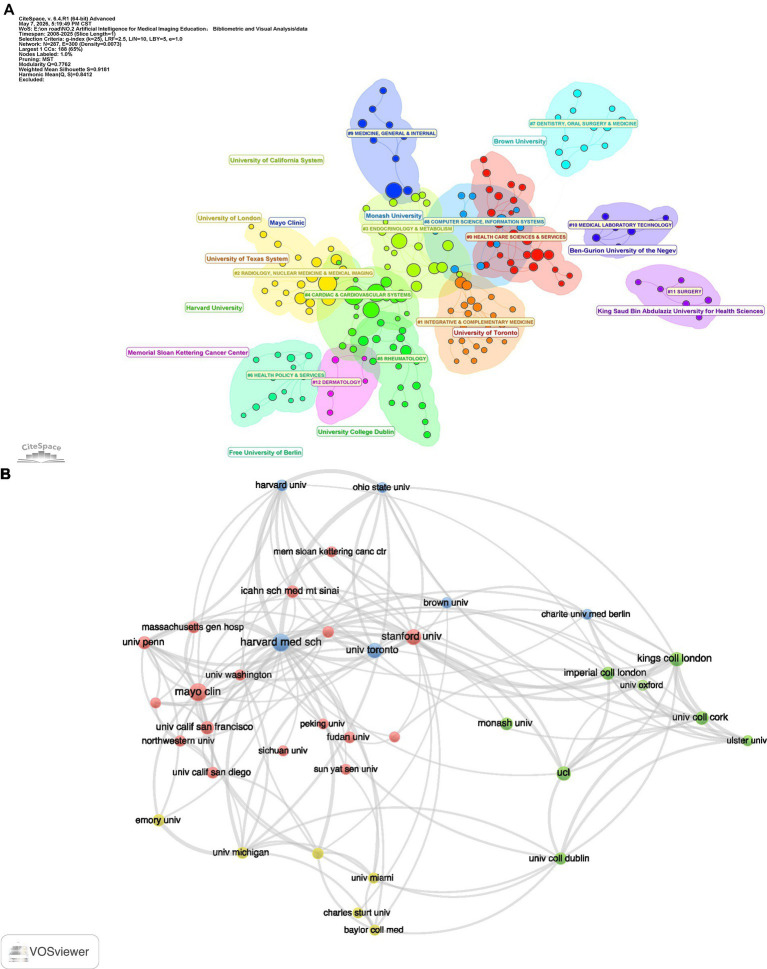
Visualization of distribution by institutions. **(A)** Visualization of institutional collaboration and citation networks. **(B)** Bibliographic coupling network of institutions.

Figure 3A shows the thematic structure of AI medical imaging education through institutional co-occurrence clusters. Three core modules dominate the network: Cluster #0 (red, “radiology application”) centers on Stanford University; Cluster #1 (orange, “usmle step”) features Harvard University and affiliates; and Cluster #2 (yellow-green, “training standard requirement”) includes UCL and Mount Sinai. These clusters form a clinical-educational triad with dense interconnections. Peripheral clusters, such as #4 “integrative oncology” and #5 “new computational age,” complement this core but demonstrate sparser network integration.

Institutional citation patterns exhibit distinct geographic clustering, with North American, European, and Asian institutions forming separate but interconnected groupings. Figure 3B presents the bibliographic coupling network of institutions in AI for medical imaging education, revealing differentiated citation patterns across geographic regions. Six clusters are identified, of which three constitute major groupings. The red cluster occupies the network core, comprising major US medical schools (Mayo Clinic, Harvard Med Sch, Yale Sch Med) that display the largest nodes and densest internal connectivity. The blue cluster forms a European grouping (Kings Coll London, Univ Coll Cork, European Soc Med Imaging Infor) with dense internal coupling but sparse connections to the red cluster. The green cluster occupies a structural bridge position, linking Western institutions (Johns Hopkins Univ, UCL, Monash Univ) with Asian universities (Peking Univ, Fudan Univ, Sun Yat-sen Univ). Smaller peripheral clusters, including the yellow group (Stanford Univ, Imperial Coll London) and the cyan group (MIT, Harvard Univ), exhibit weaker coupling with the core clinical cluster. North American academic medical centers occupy central positions, European institutions form regionally distinct groupings, and Asian universities maintain coupling ties to the core through intermediary institutions.

### 3.5 Distribution of authors

In this section, the author statistics are based on the search results from WoS (search query TS = (“artificial intelligence” OR “AI”), TS = (“medical imaging” OR “medical image*”), and TS = (“educat*” OR “teach*”)), with a total of 577 documents and 3,739 authors.

**Figure 4:**
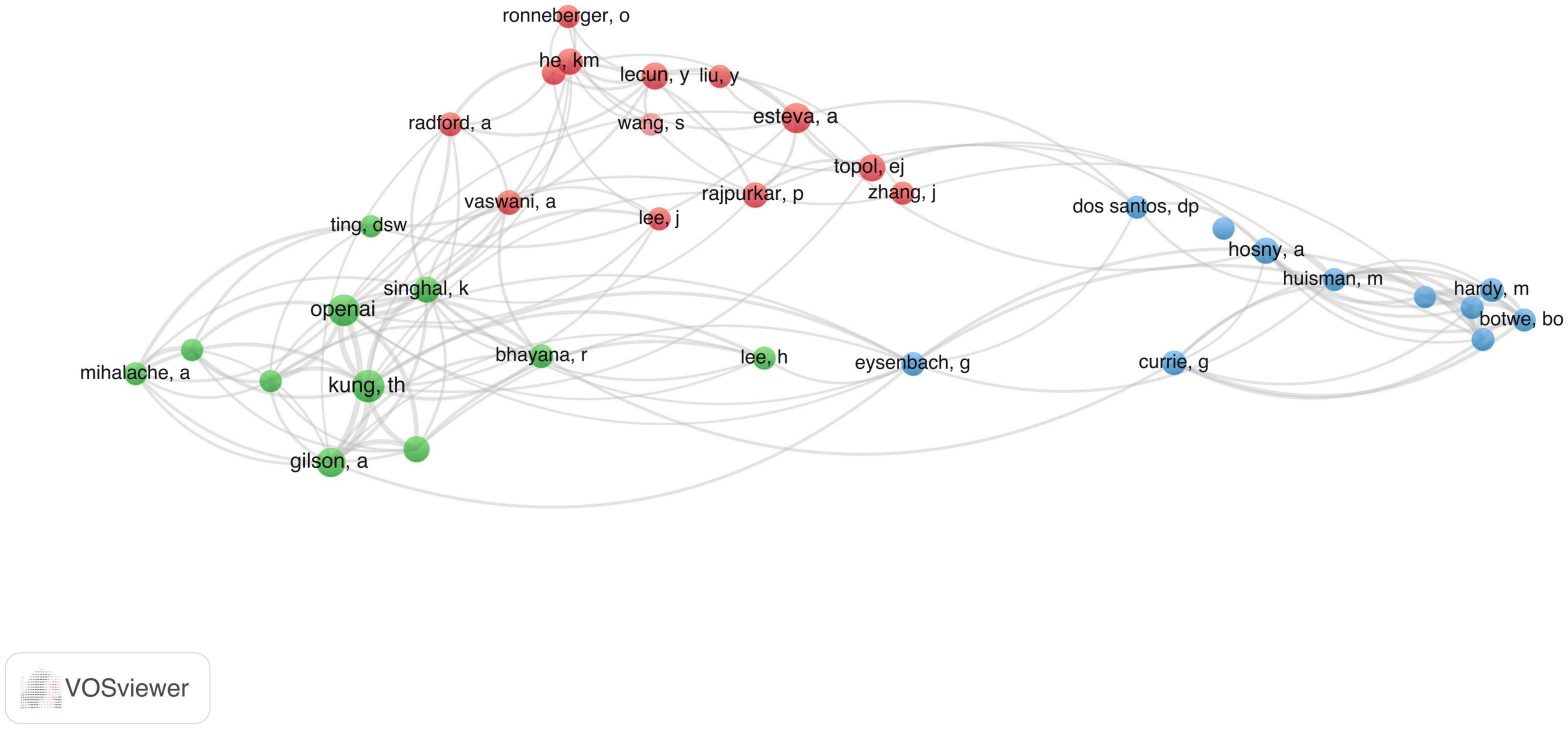
Author co-citation network of AI in medical imaging education. Node size indicates co-citation frequency. Colors denote distinct clusters, representing different research communities within the field.

The authors’ co-citation network revealed three distinct clusters (Figure 4). The red cluster features foundational computer scientists such as He KM (ResNet architecture) and LeCun Y (deep learning), representing the computer vision and deep learning domains (35, 36). Large language model technologies (exemplified by ChatGPT and GPT-4) are positioned between the red and blue clusters (12, 13). The green cluster centers on clinical AI application researchers such as Esteva A (dermatology AI) and Topol EJ (digital medicine) (8, 37), while the blue cluster includes scholars in medical informatics and education, exemplified by Kung TH (ChatGPT in medical education) and Gilson A (AI clinical reasoning) (12, 13).

Network topology metrics reveal a compact structure (average shortest path length 1.81, diameter 4.00). The red and green clusters exhibit dense inter-cluster connections, with numerous edges linking clinical application and computational methodology nodes. The blue cluster occupies a relatively peripheral position relative to the red-green axis but exhibits strong internal co-citation density, with sparse external connections to the core clusters. Overall, the red and green clusters form the network core with dense interconnections, while the blue cluster maintains strong internal coupling despite fewer external ties.

### 3.6 Distribution of journals

In our assessment of AI for medical imaging education, we identified high-output, high-impact journals by a bibliometrics online analysis platform. Journal influence is commonly assessed by impact factor (IF) and Journal Citation Reports (JCR) quartiles. Within each discipline, JCR ranks journals by their prior-year IF, stratifying them into four equal tiers: Q1 (top 25%), Q2 (25th–50th percentile), Q3 (50th–75th percentile), and Q4 (bottom 25%). A total of 319 journals have published research on AI in medical imaging education, with the top 10 most productive journals exclusively originating from five countries (Table 3). Notably, the journals with the highest representation are “BMC Medical Education,” “Insights into Imaging,” and “Academic Radiology,” with 19, 15, and 13 articles published, respectively. Among the top 10 journals, two are in Q1, eight in Q2. Geographically, the top 10 journals are distributed across five countries, with Switzerland contributing the largest share (three journals). The United Kingdom, Canada, and the United States each host two journals, while Germany accounts for one. Thematically, they span radiology/imaging journals (41 articles, 35.7%), medical education (32 articles, 27.8%), and multidisciplinary technology outlets (35 articles, 30.4%), while digital health representation remains limited (7 articles, 6.1%).

**Table 3:** Top 10 most productive journals and their impact metrics.

Rank	Journal	Publications	IF	JCR	Country
1	BMC Medical Education	19	3.6	Q2	United Kingdom
2	Insights into Imaging	15	4.8	Q1	Germany
3	Academic Radiology	13	3.9	Q2	United States
4	Diagnostics	13	3.6	Q2	Switzerland
5	JMIR Medical Education	13	3.0	Q2	Canada
6	Frontiers in Medicine	11	3.9	Q2	Switzerland
7	Applied Sciences-Basel	8	2.7	Q2	Switzerland
8	Scientific Reports	8	3.8	Q2	United Kingdom
9	IEEE Access	8	3.4	Q2	United States
10	Journal of Medical Internet Research	7	5.8	Q1	Canada

Impact factors and JCR quartiles reported in this table were taken from the 2024 JCR release.

**Figure 5:**
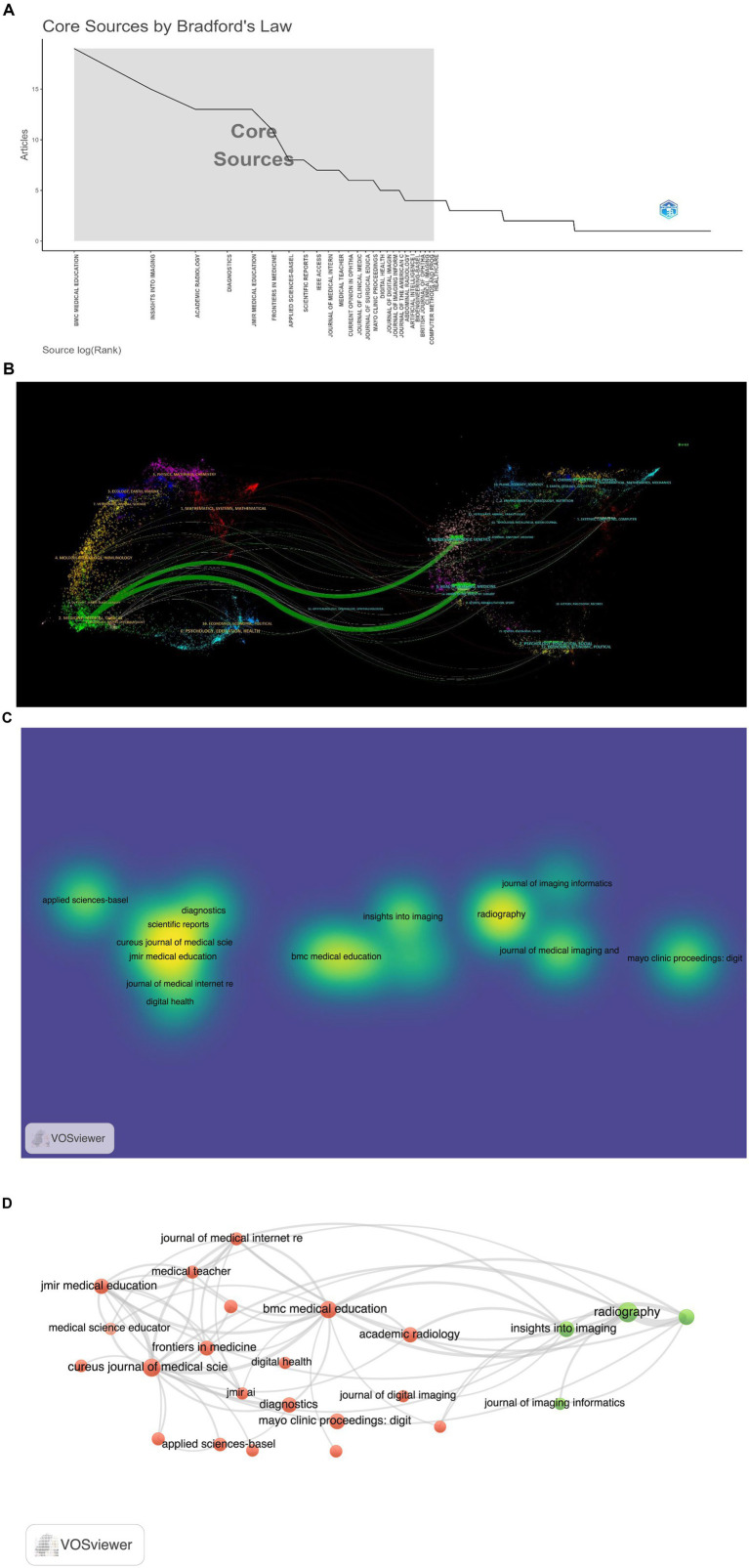
The analysis of journals. **(A)** The plot of Broadford’s law. Citing journals (left); cited journals (right). **(B)** Dual graph overlay of journals. **(C)** Journal visualization network diagram. **(D)** The journals’ collaboration and citation network diagram.

Bradford’s Law of Scattering was applied to identify core journal clusters, revealing a distribution that deviates from the idealized 1:n:n^2^ ratio (38, 39, 41). While the first five journals accounted for the core zone (Zone 1) (40), they exhibited a bimodal structure bifurcated between medical education (BMC Medical Education, JMIR Medical Education) and radiology/imaging venues (Insights into Imaging, Academic Radiology, Diagnostics) (41), deviating from the unimodal concentration typical of single-discipline fields (Figure 5A). Notably, the distribution displayed a stepwise descent with a secondary plateau at eight articles (Applied Sciences-Basel, Scientific Reports, IEEE Access), diverging from the ideal exponential ratio (1,n:n^2^) expected in traditional Bradford distributions. These open-access platforms occupied the Zone 2–Zone 3 boundary (related-to-peripheral transition).

Figure 5B applies the Chen & Leydesdorff dual-map overlay, with source journals on the left and target journals on the right (42). The citing portfolio (left) clusters primarily into Medicine, Medical, Clinical (#2), Psychology, Education, Health (#6), and Mathematics, Systems, Mathematical (left #1), covering clinical medicine, education, and computational science categories. The cited knowledge base (right) concentrates on Health, Nursing, Medicine (#5), Molecular, Biology, Genetics (#8), and Systems, Computing, Computer (right #1). The dominant citation flows (thick green streams) bridge the clinical-educational interface toward the health-medicine core. No single journal acts as the exclusive hub on both sides.

Figure 5C, the Journal Visualization Network Diagram, shows that brightness in Density Visualization merely reflects the co-occurrence frequency between journal names and the topic. It does not represent academic impact. At the center, BMC Medical Education, Insights into Imaging, and the Diagnostics–Applied Sciences-Basel cluster appear to constitute the brightest core. Brightness decreases outward: the yellow–green transition zone shows moderate intensity; the green–blue rim appears faint. This multimodal gradient distributes high density across three overlapping regions (medical education, clinical imaging, and general diagnostics). The yellow–green zone primarily includes Academic Radiology, Medical Teacher, Healthcare, and IEEE Transactions on Medical Imaging. Meanwhile, Digital Health, Journal of Medical Internet Research, IEEE Journal of Biomedical and Health Informatics, and ophthalmology titles such as British Journal of Ophthalmology tend to concentrate in the green–blue periphery.

Figure 5D scales node size to occurrence frequency: BMC Medical Education appears as the dominant central node, followed by Insights into Imaging, while other journals are markedly smaller. Nodes are auto-clustered into four major color groups: a green medical-education–radiology cluster (BMC Medical Education, Academic Radiology, Medical Teacher), a red imaging–bioengineering group (Insights into Imaging, Bioengineering-Basel), a yellow digital-health subset (JMIR Medical Education, Healthcare), and a pink JMIR-related group (Digital Health, Journal of Medical Internet Research), with smaller cyan, orange, and purple nodes scattered at the periphery representing diagnostics-technology, multidisciplinary, and surgical–ophthalmology interests. BMC Medical Education remains the largest. Links appear slightly more concentrated within clusters than between them, with intra-cluster link density exceeding inter-cluster connections. The network exhibits a fragmented yet multi-polar pattern.

### 3.7 Keyword analysis

Keyword analysis was conducted to delineate research hotspots and emerging frontiers in AI for medical imaging education.

Table 4 lists the 10 most frequent keywords. “artificial intelligence” ranked first with 270 occurrences and a Total Link Strength (TLS) of 526, followed by “medical education” with 78 occurrences and a TLS of 206. “machine learning” (70 occurrences, TLS 172), “deep learning” (61 occurrences, TLS 129), and “chatgpt” (60 occurrences, TLS 195) also featured prominently. Notably, “ai” (41 occurrences, TLS 124) is the abbreviation for “artificial intelligence,” and its considerable frequency demonstrates the prominence of AI-related research in this domain. Additionally, “large language models” (36 occurrences, TLS 113) represents the broader technological category encompassing “chatgpt,” while “radiology” (33 occurrences, TLS 67) and “medical imaging” (26 occurrences, TLS 55) anchor the specific clinical contexts. This dual presence of technical AI terminology and pedagogical keywords documents a distribution spanning computational innovation and medical education, with radiology and medical imaging serving as the primary clinical anchors.

**Table 4:** The 10 most frequent keywords and Their TLS.

Rank	Keyword	Occurrences	TLS
1	artificial intelligence	270	526
2	medical education	78	206
3	machine learning	70	172
4	deep learning	61	129
5	chatgpt	60	195
6	AI	41	124
7	large language models	36	113
8	radiology	33	67
9	education	31	65
10	medical imaging	26	55

**Figure 6:**
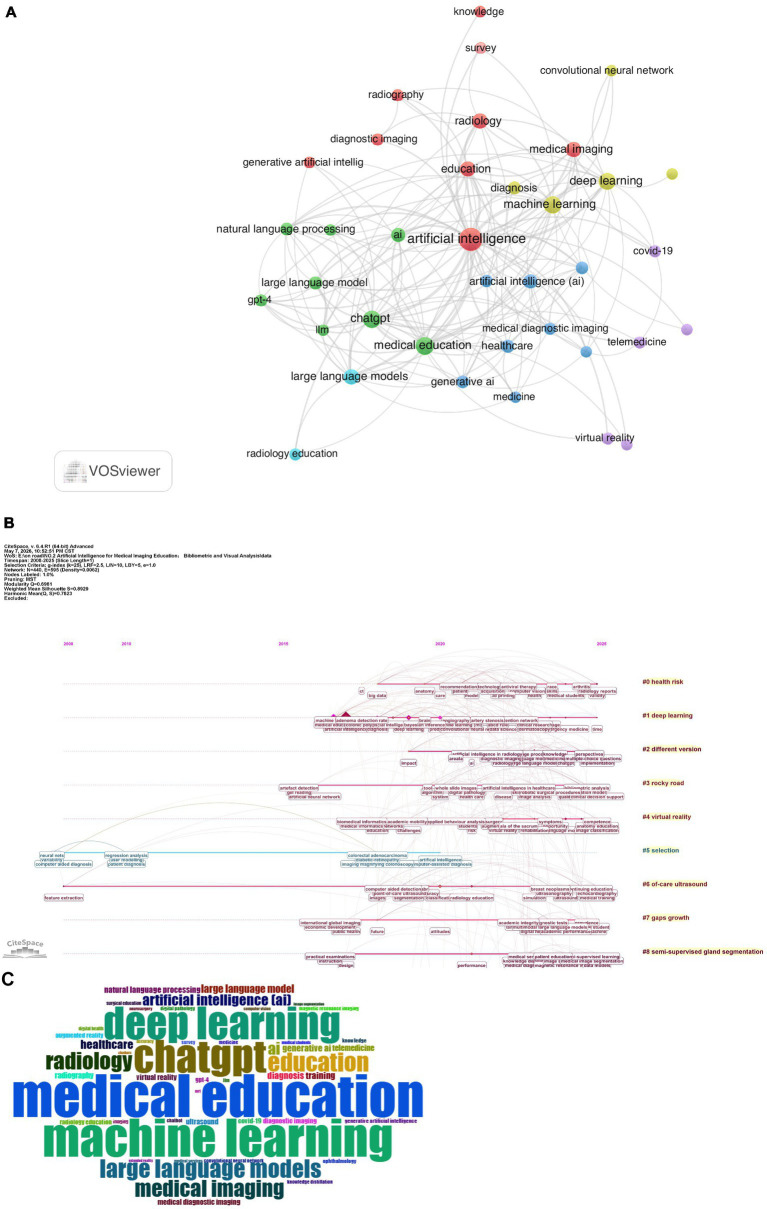
The analysis of keywords. **(A)** Keyword co-occurrence network. **(B)** Keyword cluster timeline view. **(C)** Word cloud based on WoS.

Figure 6A illustrates a keyword co-occurrence network in which node size reflects occurrence frequency and edges represent co-occurrence ties. The blue cluster centers on “artificial intelligence” as the dominant node, surrounded by “machine learning”; the green cluster groups “deep learning,” “medical imaging,” “segmentation,” “neural networks,” and “classification”; the yellow cluster links “large language models,” “chatgpt,” “gpt-4,” and “generative ai”; the red cluster connects “education,” “medical education,” “health-care,” and “challenges”; and the purple cluster comprises “virtual reality,” “augmented reality,” and “radiology education.” Dense edges converge on “artificial intelligence,” reflecting its central position connecting educational applications, large language model technologies, and medical imaging methodologies.

Figure 6B employs horizontal bands to indicate the years in which new keywords were co-cited within each cluster: a continuous segment means fresh terms were added in that year, whereas a gap signals none. The band for cluster #1 “artificial intelligence” runs continuously from approximately 2015 to 2025, successively incorporating education, medical education, deep learning, and challenges. Likewise, the band for cluster #3 “machine learning” spans from 2015 onward with intermittent continuity, introducing classification, segmentation, and neural networks. Cluster #4 “medical imaging” also persists throughout the timeline, albeit with gaps, successively adding diagnosis, radiology, and images. Meanwhile, clusters #0 and #2 (“large language model” and “large language models”) emerge later, around 2022–2023, rapidly accumulating chatgpt, generative ai, and natural language processing. Because the bands for clusters #1, #3, and #4 substantially overlap in time, these clusters remained active concurrently as the foundational research hotspot, whereas the recent emergence of LLM-related clusters (#0, #2) coincides with conversational AI applications in medical education. This temporal divergence, evident in the post-2022 emergence of LLM-related clusters (Figure 6B, coinciding with the release of ChatGPT (12, 13)), corresponds to a broadening of methodological repertoire, rather than a linear progression from foundational machine-learning algorithms.

Figure 6C Word Cloud visually presents the key themes in “Artificial Intelligence for Medical Imaging Education.” Among them, “medical education” stands out as the most prominent keyword with the largest font size. Following closely are “machine learning,” “deep learning,” and “ChatGPT,” which appear in larger fonts, indicating high occurrence frequency. Additionally, “large language models,” “radiology,” and “medical imaging” are displayed in larger fonts, indicating high frequency in the dataset.

Although terms such as “natural language processing,” “convolutional neural network,” “radiology education,” “digital pathology,” “telemedicine,” “image segmentation,” “metaverse,” “augmented reality,” and “virtual reality” appear in smaller fonts, their presence reflects varied research topics including advanced imaging algorithms, subspecialty education, and immersive learning environments. Overall, visualization shows both the central position of medical education and the presence of machine learning, deep learning, LLMs, and radiological imaging technologies on medical training and diagnostic pedagogy.

### 3.8 Highly cited reference analysis

**Figure 7:**
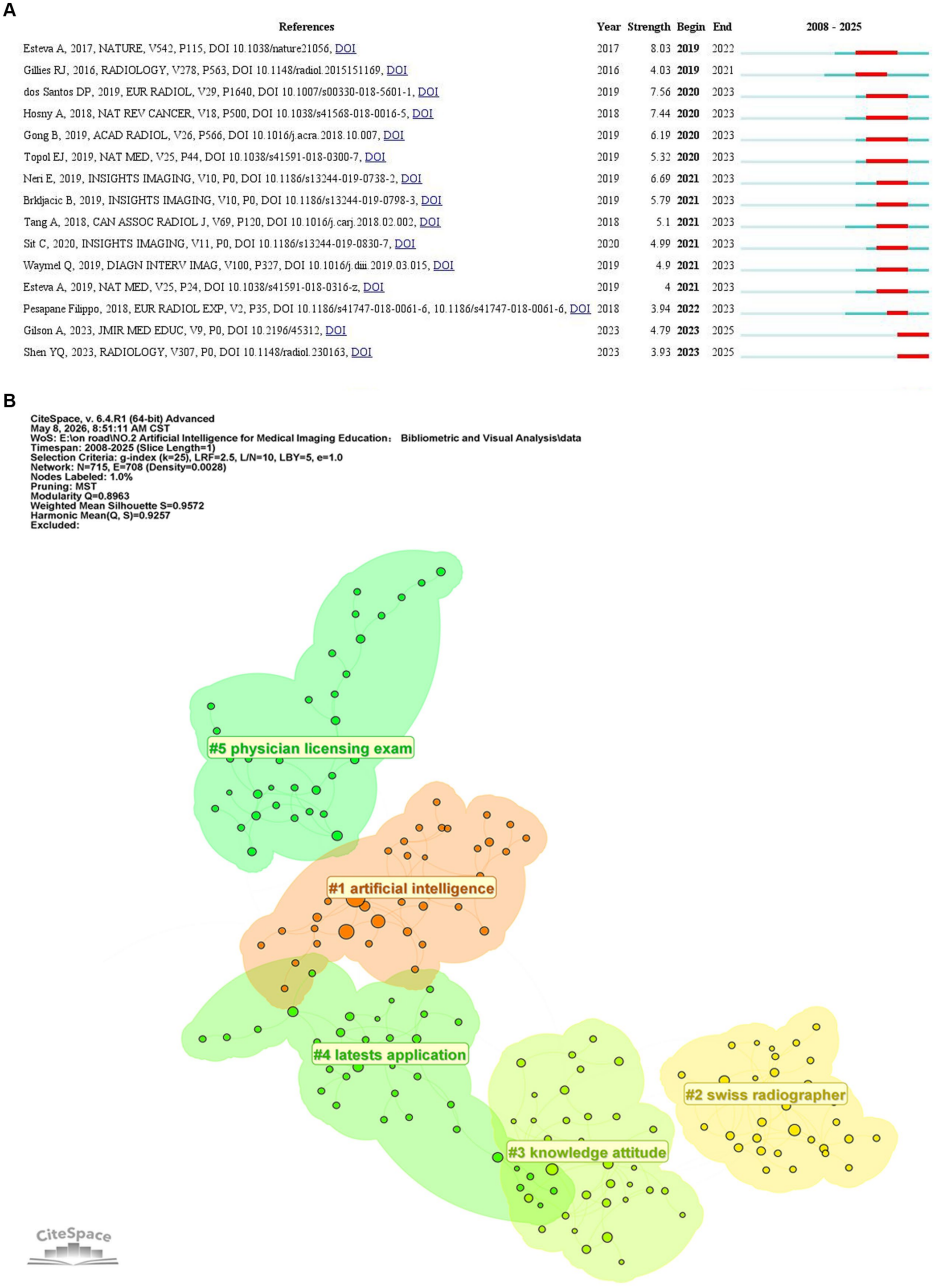
Exploration of references regarding AI for medical imaging education. **(A)** Top 15 references the most notable citation surges. **(B)** Clustered co-citation network of references.

Table 5 presents 10 highly cited references grouped into four interlocking dimensions. Studies on LLM performance validation and risk assessment reveal that ChatGPT achieves USMLE passing competency, while demonstrating multi-scenario utility in medical education (10, 12–14). Specifically, these assessments also identify critical hallucination risks (10, 17). Separately, research surveys medical students’ attitudes toward AI integration in radiology curricula (20). Clinical diagnostic benchmarking establishes rigorous standards for AI in medicine. Research achieves dermatologist-level accuracy for skin cancer classification and establishes comprehensive medical QA benchmarks, yet shows persistent clinical reasoning limitations (8, 26). Technical implementation frameworks provide the foundational architecture for clinical deployment. These studies delineate deep learning principles for oncologic imaging and examine clinical workflow integration across radiology and pathology (9, 37). Ethical governance and risk mitigation address the societal and academic implications of these technologies. Research explores AI’s dual-use potential and highlights academic integrity threats from fabricated references, urgently advocating for standardized ethical frameworks (10, 14, 43). Collectively, these works range from algorithm validation to clinical implementation, with citation patterns examined in Figures 7A,B.

**Table 5:** Top 10 highly cited references.

Rank	Key articles	Cited	Core findings
1	Kung TH, 2023, PLOS DIGIT HEALTH (12)	89	This study provides the first evidence that ChatGPT achieved performance at or near the USMLE passing threshold without specialized medical training, while generating clinically insightful explanations, thereby offering proof of concept for LLM-assisted medical education and clinical decision-making.
2	Gilson A, 2023, JMIR MED EDUC (13)	60	This study confirms that ChatGPT attained passing scores on USMLE Step 1 and Step 2 with logically rigorous and contextually rich explanations, significantly exceeding the performance of earlier language models, thus validating its potential as an interactive medical education tool.
3	Sallam M, 2023, HEALTHCARE-BASEL (43)	37	This PRISMA-compliant review examines the dual impact of ChatGPT across health care, balancing transformative potential against critical ethical risks, and underscores the need for standardized ethical frameworks.
4	Hardy M, 2019, BRIT J RADIOL (56)	30	This study fills the gap in AI impact literature regarding radiographers (as distinct from radiologists) by assessing automation potential across imaging workflows, thereby delineating opportunities for role expansion into patient-facing care, cross-modality education, and AI-assisted reporting.
5	dos Santos DP, 2018, EUR RADIOL (20)	25	This survey study (n = 263) demonstrates that undergraduate medical students, contrary to media-induced anxiety, do not fear AI replacing radiologists but instead recognize its transformative potential for radiology and strongly advocate integrating AI into medical curricula.
6	Singhal K, 2023, NATURE (26)	24	This study introduces MultiMedQA, a comprehensive benchmark for medical question answering paired with a multi-dimensional human evaluation framework, demonstrating that while Med-PaLM achieves breakthrough performance on objective metrics (67.6% accuracy on MedQA), human evaluation shows significant persistent limitations in clinical reasoning capabilities.
7	Topol EJ, 2019, NAT REV (37)	23	This review systematically shows deep learning applications across three domains, namely clinical image interpretation, health systems workflow, and patient self-management; critically evaluates associated limitations (bias, privacy, transparency); and cautions against the technology’s dual-edged impact on the patient-doctor relationship.
8	Sit C, 2020, INSIGHTS IMAGING (46)	19	This study shows that AI concerns deter nearly half of UK medical students from pursuing radiology careers, shows the critical deficit in AI education (only 9% receiving optional training), and underscores the urgent need to expand medical curricula with realistic AI capabilities and limitations to sustain the radiology workforce.
9	Esteva A, 2017, NATURE (8)	19	This study trains a deep convolutional neural network on 129,450 clinical images spanning 2,032 diseases, achieves diagnostic accuracy comparable to 21 board-certified dermatologists across two critical skin cancer classification tasks, and demonstrates the potential for mobile AI to deliver low-cost, universal access to vital skin cancer screening.
10	Hosny A, 2018, NAT REV CANCER (9)	19	This Opinion article systematically reviews the technical principles and applications of deep learning-based AI methods in oncologic imaging analysis, explores the transition from qualitative to quantitative radiographic assessment, and examines critical challenges impeding clinical implementation.

The numbers preceding “Key Articles” are reference numbers.

Figure 7A highlights the 15 references with the most pronounced citation bursts, pinpointing the windows during which research attention surged. Dos Santos DP et al. (2018) led with a burst strength of 6.44, active from 2021 to 2023, while Esteva A (2017) (6.3), Hosny A (2018) (5.14), Topol EJ (2019) (5.07), and Gong B (2018) (5.07) exhibit high-intensity bursts concentrated in 2019–2023 (44). Research on citation patterns suggests that such pronounced bursts often characterize papers introducing transformative methodologies or addressing timely disciplinary concerns (44). Gillies RJ (2016) (3.32) and Gulshan V (2016) (3.32) triggered earlier bursts from 2019 to 2021, whereas Gilson A (2023) (4.48) prolongs the surge into 2023–2025. Collectively, Figure 7A shows that the most explosive citation peaks cluster in 2019–2023, offering bibliometric support for the highly cited references listed in Table 5.

Figure 7B presents a co-citation cluster network visualizing the thematic structure of AI in medical imaging education, with clusters labeled by extracted terms and sized by citation frequency. At the structural center, cluster #0 (artificial intelligence) occupies the geometric center as the largest node, with “algorithm” (255 co-citations, silhouette 0.995) as its central term and highly cited references to ResNet, LLM performance benchmarks, and ChatGPT reliability assessments (26, 27). Clusters #1 (generative artificial intelligence) and #2 (deep learning) occupy core positions adjacent to cluster #0. Cluster #1 focuses on technical innovations such as deep learning, neural networks, and AI education; cluster #2 centers on systematic review, diagnostic radiology, and ChatGPT, with distinct yet adjacent positions. Cluster #3 (medical student) forms a distinct yet connected nucleus alongside cluster #8 (French radiology community), positioned between the core computational clusters and the periphery. Smaller but tightly connected nodes, including cluster #4 (medical image), #5 (generative AI), #6 (segmentation), #9 (education), and #10 (convolutional neural networks), fill intermediate positions, while peripheral clusters #12 (radiology, radiation oncology) and #7 (COVID-19 screening) occupy the outer edges.

Meanwhile, intermediate clusters such as #4 (medical image, systematic review), #5 (generative AI), #6 (segmentation), #9 (education), and #10 (convolutional neural networks) occupy positions between the core and the periphery, maintaining moderate co-citation ties. This core–intermediary–periphery architecture organizes nodes across computational, educational, and clinical domains.

### 3.9 Cross-database validation

As shown in Table 6, the cross-database comparison of the most productive countries further demonstrates the consistency of findings between WoS and Scopus. When ranked by publication count, all five countries maintained identical ranking positions across both databases: the United States and China were identified as the top two contributing nations, followed by the United Kingdom with markedly lower output, corroborating the ranking patterns in Table 1. Proportional contributions showed similar patterns despite absolute number differences attributable to database coverage variations (USA: 28.8% vs. 19.8%; China: 19.9% vs. 16.8%). This ranking concordance is consistent with the dual-polar global structure identified in the primary WoS analysis.

**Table 6:** Cross-database comparison of productive countries.

Rank	Country	WoS (*n* = 577)	Scopus (*n* = 369)	Concordance
1	USA	166, 28.8%	73, 19.8%	Yes
2	China	115, 19.9%	62, 16.8%	Yes
3	UK	31, 5.4%	25, 6.8%	Yes

Rankings are based on publication count.

**Figure 8:**
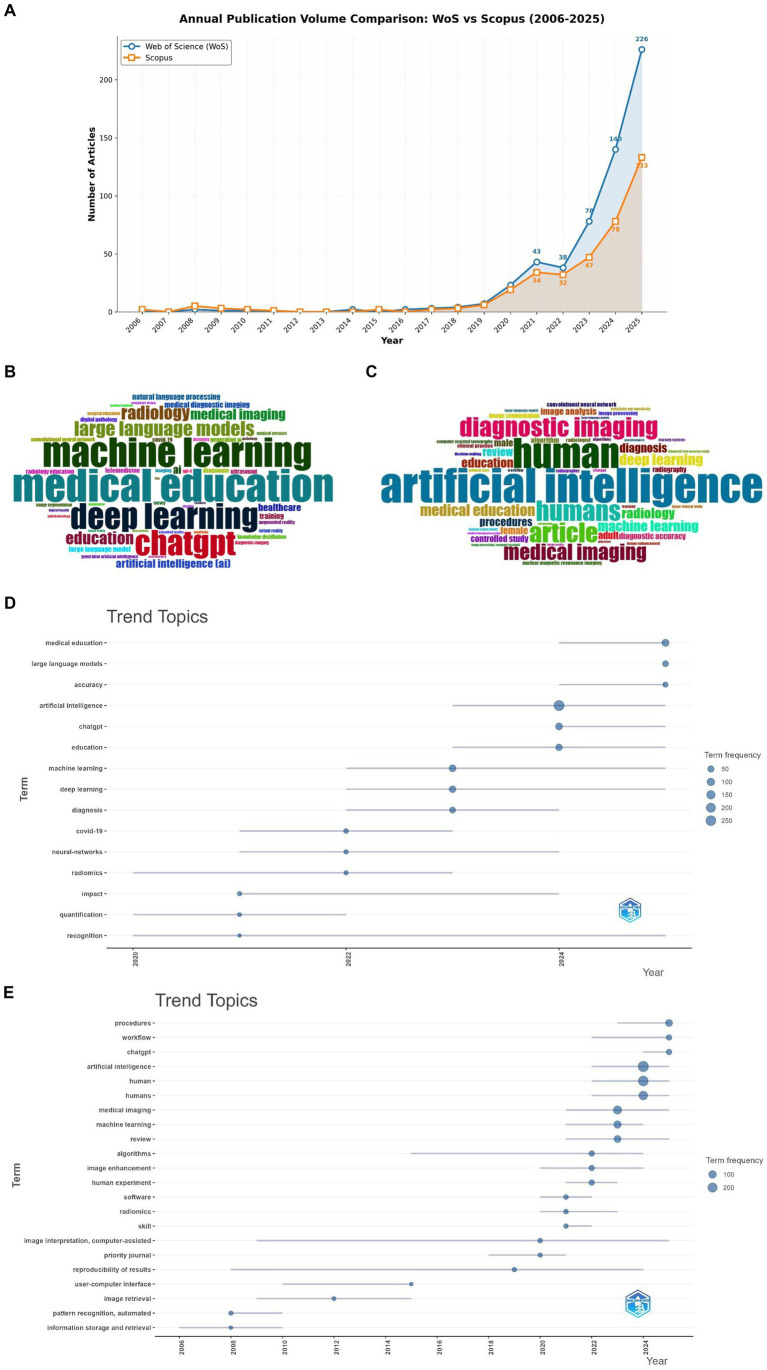
Cross-database validation between WoS and Scopus. **(A)** Annual publication volume comparison between Web of Science and Scopus (2006–2025). **(B)** Word Cloud based on WoS. **(C)** Word Cloud based on Scopus. **(D)** Trend topics based on WoS. **(E)** Trend topics based on Scopus.

Annual publication trends demonstrated remarkable synchronization between the two databases (Figure 8A). After applying identical eligibility criteria, WoS yielded 577 publications and Scopus yielded 369. Both databases exhibited similar four-phase growth trajectories: negligible activity during 2006–2019 (mean < 3 articles/year), synchronous initiation in 2020 (23 vs. 19 articles), acceleration in 2021 (43 vs. 34 articles), exponential growth in 2022 (38 vs. 32 articles), and continued expansion through 2024 (140 vs. 78 articles). Notably, in 2025, WoS indexed 226 articles compared to 133 in Scopus. Absolute volume differences reflect database coverage variations. The Spearman correlation coefficient of 0.97 (*p* < 0.001) shows directional consistency in publication trends across both databases. In the keyword analysis (Figure 8B), both databases identified “artificial intelligence,” “medical education,” “radiology,” “deep learning,” “machine learning,” and “medical imaging” as the top six high-frequency keywords, with only minor variations in ranking order. Both databases also tracked the same temporal evolution in keyword usage.

Figure 8C compares keyword evolution trajectories between WoS and Scopus, revealing a post-2022 surge in generative AI-related terms. Notably, from 2022 to 2023, keywords such as “ChatGPT,” “GPT-4,” and “generative AI” rapidly gained prominence in both databases. Word cloud analysis showed similar keyword distributions between WoS and Scopus, with both databases identifying “artificial intelligence,” “deep learning,” and “medical education” as dominant terms. This alignment confirms that both databases captured comparable keyword distributions within the field.

Figures 8D,E display the keyword evolution timeline (2015–2025) from WoS and Scopus, revealing a consistent developmental trajectory across both databases. From 2015 to 2021, both databases identified “deep learning,” “neural networks,” “medical imaging,” and “convolutional neural network” as dominant keywords. In 2022–2023, both databases tracked the emergence of generative AI-related keywords, including “ChatGPT,” “GPT-4,” and “generative artificial intelligence.” Both databases traced the transition from early technical foundations to the current generative AI era. Notably, both databases tracked the concurrent emergence of generative AI applications and medical imaging education keywords, documenting an observable, cross-database trend. Furthermore, both databases identified 2022–2023 as the critical period for the emergence of LLM-related keywords, indicating the influence of large language models and generative AI on medical imaging education research. These findings are based on bibliometric data, which provide quantitative indicators of research trends, and any interpretation of methodological trends should be supported by complementary qualitative analyses.

Through a triple-validation strategy encompassing annual publication trends, keyword distributions, and thematic evolution trajectories, Figure 8 collectively documents the consistency of findings across independent indexing systems. This cross-database concordance (577 articles in WoS and 369 in Scopus) reduces database-specific bias and documents the observed exponential growth and generative AI transition in medical imaging education research.

## 4 Discussion

We begin by clarifying the epistemological scope of this bibliometric analysis. The Discussion is organized around two tiers of findings: descriptive patterns directly supported by bibliometric data, and interpretive inferences positioned as hypothesis-generating starting points for future empirical research.

The post-2022 surge (Figure 1B) might coincide with several interrelated developments. First, the post-2023 surge temporally overlaps with the emergence of generative AI technologies (ChatGPT released in November 2022; GPT-4, 2023). However, acknowledging typical academic publication lags, the 2025 peak may reflect accumulated research momentum and sustained investigator interest, though alternative explanations (such as post-pandemic academic productivity recovery and open-access publishing expansion) cannot be excluded. This expansion may partially reflect the integration of conversational AI tools into research agendas (Table 4; Figure 6B), without establishing direct causation. Unlike earlier algorithmic applications primarily serving as diagnostic aids, LLM-based implementations enable interactive, dialog-based learning environments (12, 13). This functional distinction shifts from pattern recognition to clinical reasoning support, which may account for the observed broadening of research focus without implying a decline in foundational technical research. Second, this growth may reflect a diversification of research dimensions to encompass ethical governance and stakeholder perceptions (e.g., student attitudes, curriculum integration challenges), alongside continued foundational technology research. These interpretations remain hypothetical and are not mutually exclusive; the surge might also reflect the influence of external factors, including the post-pandemic recovery of global academic productivity and the expansion of open-access publishing policies.

Table 1 shows that Germany exhibited the highest average citations per article (46.40) among the top 10 productive countries, despite ranking fourth in total output (27 articles). This pattern likely reflects two complementary factors. First, statistical effects of small sample sizes render national averages susceptible to skewing by a few highly cited reviews; with only 27 publications, Germany’s mean citation rate is disproportionately influenced by outlier papers. By contrast, Canada, with a similarly limited output (20 articles), achieved a lower average (35.00), suggesting that Germany’s advantage might additionally stem from structural variations in publication types (for example, high-impact reviews vs. original research) rather than intrinsic national quality differences. Second, network topology factors may contribute: Germany’s high MCP rate (48.1%) reflects extensive international collaboration (45), and its boundary position between North American and European clusters (Figure 2B) may confer broader citation visibility. Thus, Germany’s high average citations probably reflect the combined influence of small-sample statistical effects and advantageous network topology.

Table 2 shows that the University of California System (19 articles, centrality 0.01) exhibited a disparity between publication volume and network influence, in contrast to high-output institutions with strong network centrality, such as Harvard University Medical Affiliates (29 articles, centrality 0.08). Figure 3B suggests that this institution might function primarily as a peripheral node within a specific cluster, with limited cross-cluster connections. Its collaborative network appears largely confined to specific regional or thematic boundaries rather than establishing extensive international cross-domain connections. This “high-volume, low-cross-cluster connectivity” pattern stands in stark contrast to institutions such as Imperial College London (13 articles, centrality 0.07), which appear to connect transcontinental clusters. These observations suggest that, in emerging interdisciplinary fields, publication productivity might not necessarily correlate with network influence: although the University of California System demonstrates considerable output, its distribution might rely on indirect pathways.

Based on Bradford’s Law analysis presented in Table 3 and Figure 5A, the journal distribution in this field deviates from the ideal BradfordIan pattern (1:n:n^2^) characteristic of single-discipline fields. This deviation likely stems primarily from the field’s bimodal structure: knowledge production is simultaneously anchored in traditional medical education channels and imaging technology innovation platforms, dispersing publication output across two distinct thematic clusters within the core zone. Within Zone 1, the output gap between the leading journal (BMC Medical Education, 19 articles) and the second-ranked (Insights into Imaging, 15 articles) is modest (approximately 26%), while journals ranked 3rd to 5th each contributed 13 articles, forming a plateau rather than an exponential decline. Furthermore, open-access journals (e.g., Applied Sciences-Basel, Scientific Reports) appear to create a secondary peak at the Zone 2–Zone 3 boundary (8 articles each), potentially serving as channels for interdisciplinary knowledge transfer, thereby contributing to lower concentration within the core zone.

Based on keyword co-occurrence and cluster analysis (Figures 6A,B; Table 4), generative AI technology (represented by “large language models”) appears to be emerging as a research frontier in this field. Figure 6A reveals that “generative ai,” “chatgpt,” “gpt-4,” and “large language models” have formed an independent yellow cluster, positioned alongside the green cluster (representing deep learning foundations) and the red cluster (representing educational applications). The co-occurrence of these terms within this cluster shows that recent literature treats them as an interconnected semantic field, wherein scholars employ “ChatGPT” as a metonym for LLM-based educational tools while “generative AI” functions as the broader conceptual umbrella (12–14).

Furthermore, Figure 6B suggests that these LLM-related clusters (#0 and #2) emerged exclusively after 2022–2023, approximately coinciding with the release timeline of ChatGPT. This temporal divergence suggests a qualitative transition in pedagogical application: from “AI-as-instrument” (traditional deep learning for automated pattern recognition) to “AI-as-interlocutor” (LLM-driven interactive reasoning support). Unlike earlier algorithmic applications primarily serving as diagnostic aids, LLM-based implementations (Table 5) enable generative, dialog-based learning environments. This suggests that LLMs may function as a distinct category of educational tools, potentially expanding the epistemological boundaries of medical imaging education beyond passive knowledge acquisition toward active, AI-augmented clinical reasoning.

This trend is supported by quantitative metrics (Table 4), which list both “chatgpt” (60 occurrences, TLS = 195) and “large language models” (36 occurrences, TLS = 113) as high-frequency keywords, but also by the substantive content of highly cited references (Table 5). Specifically, the leading studies by Kung et al. and Gilson et al. demonstrated that ChatGPT achieved performance at or near the USMLE passing threshold without specialized medical training, providing preliminary evidence for the potential of LLM-assisted medical education and clinical decision-making (12, 13). These capabilities suggest a potential move toward interactive, intelligent pedagogical models that extend beyond traditional knowledge delivery. However, as delineated in Sallam’s PRISMA-compliant review, this transformative potential is balanced against critical ethical risks and hallucination concerns, suggesting that the integration of LLMs into medical imaging curricula requires careful consideration of both educational benefits and safety challenges (43). Despite global calls for responsible AI in healthcare, our bibliometric analysis reveals a keyword-level underrepresentation of ethics-related terminology—terms such as “ethics,” “safety,” “privacy,” and “bias” were absent from high-frequency keyword clusters in both WoS and cross-database analyses, suggesting that within the keyword landscape, AI ethical considerations occupy a peripheral position relative to technical and educational themes. This observation is limited to term-frequency patterns and does not preclude substantive ethical discussions in article full texts, which would require full-text thematic analysis to assess comprehensively.

Thus, AI medical imaging education is expanding toward LLM-integrated clinical decision support. This evolution offers new technical possibilities for residents’ differential diagnosis training; however, it also involves issues of technical reliability and educational integration strategies that require further exploration. Building upon these identified risks and the observed ethics gap in keyword distributions, we tentatively interpret the challenges of the integration of these technologies as extending beyond merely technical domains to encompass a potential mismatch between technological development and educational preparedness. This tension is reflected in attitudinal surveys of medical students and radiographers (20, 46, 47) and in ethical governance analyses (43), and corresponds to the peripheral positioning of ethics-related keywords in our maps. This characterization remains an interpretive inference; it does not constitute a bibliometrically verified empirical finding. Despite achieving specialist-level performance in specific diagnostic tasks (e.g., skin cancer screening (8)), AI technologies appear to have advanced more rapidly than the readiness of educational systems. This study conceptualizes this tension through three analytically distinct yet empirically overlapping dimensions of potential mismatch (cognitive-affective, curriculum-competency, and capability-governance) as heuristic entry points informed by the qualitative concerns raised in highly cited references and resonant with the observed bibliometric patterns, acknowledging that these categories are neither exhaustive nor mutually exclusive. First, as documented in educational survey studies and attitudinal research (46, 47), a cognitive-affective mismatch is evident in the ‘displacement anxiety’ reported by nearly half of UK medical students and radiographers, deterring them from pursuing radiology careers. Comparable concerns have been documented among Canadian medical trainees (48) and Ghanaian radiographers (49). This apprehension stems not from rational assessments of technological unemployment but from a “cognitive discord” as reported in attitudinal surveys (46, 48), in which students simultaneously overestimate AI’s immediate substitution risks and underestimate its augmentation potential in human-AI collaboration. Second, a curriculum-competency mismatch might exist: while radiographers demonstrate positive attitudes toward AI, they exhibit a notable “cognitive gap” characterized by insufficient domain knowledge (47). The current curriculum structure, based on traditional medical education frameworks, might indicate gaps in fostering AI literacy, with only 9% of UK medical students receiving optional AI training, suggesting that pedagogical updates might lag behind technological developments (46). This observation aligns with competency-based education frameworks that prioritize progressive skill acquisition and adaptive learning design. Third, a capability-governance mismatch may emerge as LLMs demonstrate performance approaching passing thresholds on medical licensing examinations while exhibiting reported hallucination risks (43, 46). Despite these reported limitations, the integration of tools like ChatGPT into clinical decision support systems, implemented without commensurate critical thinking training or academic integrity protocols, might result in technology deployment that precedes adequate safety safeguards. These three dimensions highlight potential areas of concern for intervention, while recognizing that additional sociotechnical tensions (e.g., policy-resource gaps, institutional barriers) warrant parallel investigation educational.

Collectively, these three potential mismatches suggest a pattern-level interpretation: the current AI medical imaging education ecosystem might remain in a proof-of-concept phase, potentially lacking governance frameworks necessary for the transition toward systematic integration. Drawing on collaborative governance frameworks (50), these findings point to the need for future research on how technical standards, educational capacity-building, and ethical oversight can be systematically integrated, though specific implementation pathways remain to be empirically validated. Such a framework might be important to advance the field from experimental networks toward responsible, standardized clinical practice, helping to ensure that technological capabilities align with educational preparedness and ethical safeguards.

This study proposes a conceptual integrative framework as heuristic entry points, drawing on tensions documented in attitudinal surveys (20, 47, 48) and curriculum integration assessments (51), and resonates with the observed bibliometric patterns. Through convergent validation encompassing annual trends, keyword distributions, and thematic trajectories (Figure 8), the concordance between WoS and Scopus reduces potential source bias and provides convergent evidence for the observed exponential growth and generative AI transition. Beyond methodological consistency, this concordance suggests that the observed thematic patterns reflect genuine field developments rather than database-specific indexing artifacts. Despite this methodological rigor, several limitations warrant consideration.

## 5 Limitations

This study employs bibliometric visualization to analyze research on AI for medical imaging education over the past two decades. However, key limitations include: (1) restriction to two major bibliographic databases (WoS and Scopus), potentially omitting relevant studies indexed in other specialized or regional databases. Future systematic reviews in this domain may benefit from incorporating PubMed/MEDLINE for biomedical education depth, Embase for complementary European biomedical coverage, and IEEE Xplore for technical AI engineering literature, as well as specialized education databases such as ERIC. Such multi-database validation would assess whether the observed thematic trends persist across broader disciplinary indexing systems. (2) restriction to English-language literature; (3) the time-sensitive nature of database updates as of February 15, 2026, with potential marginal indexing delays for the most recent year; (4) The assessment of ethical content relies exclusively on keyword and co-occurrence frequency analysis, which captures only explicit term usage in titles, abstracts, and author keywords. It does not assess the depth or quality of ethical discussions within article full texts. Consequently, the observed underrepresentation of ethics-related terms reflects keyword-level visibility rather than a comprehensive evaluation of ethical engagement across the literature, and full-text content analysis may reveal more extensive ethical treatment than the bibliometric maps suggest. Additionally, the search strategy’s reliance on the “educat*” and “medical imaging” stems may have under-represented studies exclusively employing terms such as “radiology education,” “training,” or “curriculum” without concurrent indexing in the searched fields. Future systematic reviews in this domain may benefit from complementary search strings incorporating these alternative educational terminology to ensure more comprehensive coverage.

Despite these constraints, WoS remains a widely utilized standard resource in bibliometric research, with established indexing procedures for peer-reviewed literature (52, 53). However, claims regarding universal “gold standard” status remain contested, and the database’s coverage inevitably reflects specific disciplinary and linguistic biases (53–55). While its selection criteria aim to include high-impact research (52), this does not guarantee comprehensive representation of all relevant scholarship, particularly from non-English sources or emerging publication venues. Therefore, the dataset is considered representative of mainstream research standards in this field.

## 6 Conclusion

Drawing on bibliometric data from WoS (2006–2025, *n* = 577), validated through cross-database comparison with Scopus, this study characterizes the intellectual evolution and network architecture of AI in medical imaging education. Cross-database validation (Spearman r = 0.97, *p* < 0.001) supports a dual-polar, multi-centric production structure, with the United States (28.8%) and China (19.9%) as dominant contributors. The field exhibits exponential growth post-2022 driven by generative AI integration, alongside a bimodal journal distribution spanning medical education and imaging technology venues.

Unlike single-discipline fields where knowledge consolidates in core journals, the Bradfordian deviation observed in this study indicates that AI medical imaging education constitutes a bimodal knowledge space spanning medical education and imaging technology. This bifurcated structure suggests that future systematic reviews in this domain may benefit from dual-channel search strategies encompassing both educational and technical literature.

The peripheral positioning of ethics-related keywords relative to technical and pedagogical themes suggests a potential mismatch between technological development and educational preparedness. This integrative framework is proposed as a hypothesis-generating heuristic informed by highly cited primary studies, not as a bibliometrically verified empirical finding. These findings are subject to standard bibliometric constraints, including restriction to English-language literature and two major databases; empirical validation of the proposed mismatch dimensions through qualitative or mixed-methods research remains an important direction for future investigation.

## Data Availability

The original contributions presented in the study are included in the article/supplementary material, further inquiries can be directed to the corresponding author.
